# Polymeric nanoencapsulation of zaleplon into PLGA nanoparticles for enhanced pharmacokinetics and pharmacological activity

**DOI:** 10.1002/bdd.2255

**Published:** 2021-01-02

**Authors:** Yusuf A. Haggag, Ahmed Kh. Abosalha, Murtaza M. Tambuwala, Enass Y. Osman, Sanaa A. El‐Gizawy, Ebtessam A. Essa, Ahmed A. Donia

**Affiliations:** ^1^ Department of Pharmaceutical Technology Tanta University Tanta Egypt; ^2^ School of Pharmacy and Pharmaceutical Sciences Ulster University Coleraine UK; ^3^ Department of Pharmacology and Toxicology Tanta University Tanta Egypt; ^4^ Department of Pharmaceutical Technology Menoufia University Menoufia Egypt

**Keywords:** anticonvulsant activity, formulation variables, GABA, nanoparticles, optimization, oral bioavailability, pharmacokinetics, PLGA, zaleplon

## Abstract

Zaleplon (ZP) is a sedative and hypnotic drug used for the treatment of insomnia. Despite its potent anticonvulsant activity, ZP is not commonly used for the treatment of convulsion since ZP is characterized by its low oral bioavailability as a result of poor solubility and extensive liver metabolism. The following study aimed to formulate specifically controlled release nano‐vehicles for oral and parenteral delivery of ZP to enhance its oral bioavailability and biological activity. A modified single emulsification–solvent evaporation method of sonication force was adopted to optimize the inclusion of ZP into biodegradable nanoparticles (NPs) using poly (dl‐lactic‐co‐glycolic acid) (PLGA). The impacts of various formulation variables on the physicochemical characteristics of the ZP‐PLGA‐NPs and drug release profiles were investigated. Pharmacokinetics and pharmacological activity of ZP‐PLGA‐NPs were studied using experimental animals and were compared with generic ZP tablets. Assessment of gamma‐aminobutyric acid (GABA) level in plasma after oral administration was conducted using enzyme‐linked immunosorbent assay. The maximal electroshock‐induced seizures model evaluated anticonvulsant activity after the parenteral administration of ZP‐loaded NPs. The prepared ZP‐PLGA NPs were negatively charged spherical particles with an average size of 120–300 nm. Optimized ZP‐PLGA NPs showed higher plasma GABA levels, longer sedative, hypnotic effects, and a 3.42‐fold augmentation in oral drug bioavailability in comparison to ZP‐marketed products. Moreover, parenteral administration of ZP‐NPs showed higher anticonvulsant activity compared to free drug. Oral administration of ZP‐PLGA NPs achieved a significant improvement in the drug bioavailability, and parenteral administration showed a pronounced anticonvulsant activity.

## Graphical abstract

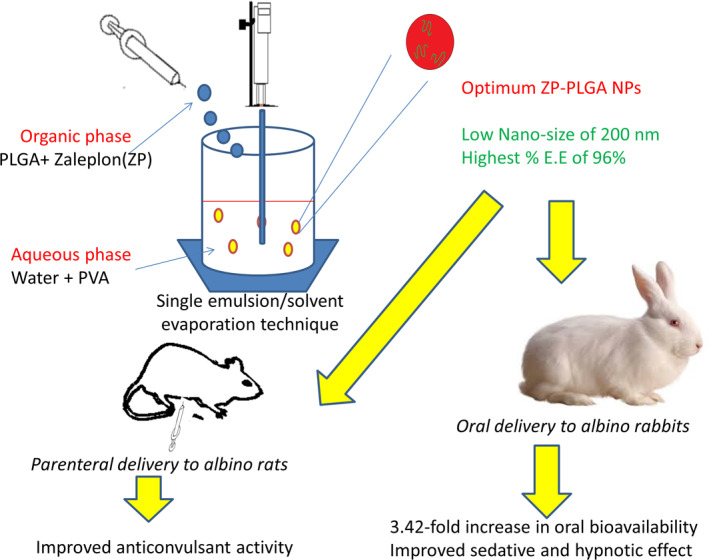

## 1 INTRODUCTION

Zaleplon (ZP) is commonly used as a sedative and hypnotic drug with potent anticonvulsant activity. The pharmacological effect of ZP is correlated to its agonist effect on the gamma‐aminobutyric acid‐A (GABA‐A) (type 1) receptor, specifically, benzodiazepine binding sites (Dooley & Plosker, 2000). Based on the biopharmaceutical classification system, ZP is categorized as a Class II drug with poor aqueous solubility and high intestinal permeability. It exhibits a relatively low oral bioavailability of about 30% (Drover, 2004). The low oral bioavailability is a result of combined poor dissolution and extensive first‐pass metabolism (Waghmare, Pore, & Kuchekar, 2008). Zaleplon is marketed in two dosage forms: tablets and capsules. However, the oral route of ZP faces numerous obstacles that hinder its oral delivery. The poor aqueous solubility slows down its dissolution with subsequent augmentation of its hepatic metabolism (Dudhipala, 2016). This leads to a disturbance in drug pharmacokinetic represented by delayed onset of action, short elimination *T*_1/2_ (1 h), and short duration of action. Therefore, ZP fails to keep its pharmacological activity reasonable, resulting in early morning awakening (Farag, El Malak, & Yehia, 2018). Increasing the dose of ZP to overcome low oral bioavailability is not recommended because it is related to typically short‐lived hallucinations (Farag et al., 2018). ZP is not among the most often prescribed sedative and hypnotic because it has a quick onset of action and very short elimination half‐life of approximately 1 h (Terzano, Rossi, Palomba, Smerieri, & Parrino, 2003). The drug exerts a better action on sleep induction rather than sleep maintenance due to its short half‐life and quick onset of action. Previous studies showed that zaleplon has a nonsignificant effect on the total sleep time and the number of awakenings (Sateia, Buysse, Krystal, Neubauer, & Heald, 2017). Different formulation approaches were used to optimize the oral delivery of ZP. These approaches vary between solid dispersion (Waghmare, Pore, & Kuchekar, 2008), micronization (Manda et al., 2018), proliposome (Janga et al., 2012), self‐nanoemulsifying powders (Janga et al., 2013), and solid lipid nanoparticles (Dudhipala & Janga, 2017).

Nanotechnology has been revolutionized the field of drug delivery and targeting regarding its supernatural qualities such as small particle size (PS), high exposed surface area, improved physical and chemical stability, and the high biocompatibility of its ingredients (Baker, 2006). Moreover, nanotechnology can optimize the solubility, dissolution, permeability, and bioavailability of several drugs regardless of their physical properties (Card & Magnuson, 2011). Nanotechnology compromises a wide range of formulation techniques such as biodegradable polymeric nanoparticles (PNPs), lipid nanoparticles, magnetic nanoparticles, polymeric micelles, liposomes, niosomes, and nanoemulsion (Brigger, Dubernet, & Couvreur, 2002).

PNPs are colloidal solid particles consisting of natural or synthetic biocompatible polymers formulated into nanoscale particles. PNPs enjoy great popularity among previously discussed nanosystems for many reasons. First of all, its ease of formulation involves only three simple steps. Second, the simplicity of adjusting and controlling its physicochemical characteristics during formulation. Also, the vast diversity of available polymers can offer a wide range of physicochemical characteristics that can be tailored depending on the desired effect (Haggag et al., 2016; Haggag & Faheem, 2015; Kamaly, Xiao, Valencia, Radovic‐Moreno, & Farokhzad, 2012; Khan, Haggag, Lane, McCarron, & Tambuwala, 2018).

PNP drug delivery systems can improve the oral bioavailability of poorly soluble drugs, sustain its biological activity, and improve drug stability (Haggag et al., 2020a). Modulation of polymer physicochemical characteristics enabled us to achieve optimal therapeutic efficacy by controlling the optimum release of a therapeutic agent for the required duration needed for attaining the desired therapeutic level in target tissues (Khan et al., 2018).

Moreover, polymeric NP systems may be able to overcome the limitations of nanoemulsions and solid lipid nanoparticles. The major drawbacks of nanoemulsion include low shelf‐life stability due to thermodynamics and Ostwald ripening and difficulty in preparation and scale‐up production. Formulation of nanoemulsions needs high concentrations of surfactant and cosurfactant necessary for improving the nanoemulsion stability (Patel, Patel, & Thakore, 2018; Yukuyama, Kato, Lobenberg, & Bou‐Chacra, 2017). However, solid lipid nanoparticles showed some other disadvantages such as the propensity of lipid oxidation and transformation, incompatibility with various active agents, and a limited drug loading efficiency (Deshpande et al., 2017; Ghasemiyeh & Mohammadi‐Samani, 2018). In contrast, polymeric nanocarriers are more stable in vivo, with high drug loading capacities, as well as controlled or triggered release of drugs (Kamaly et al., 2012). According to these unique properties, polymeric nanomaterials are well positioned in our study to provide a novel solution for ZP oral and parenteral delivery.

Oral delivery is a standard route for drug administration due to its favorable advantages of high patient compliance due to self‐administration ease. However, some physiological barriers control drug bioavailability and therapeutic activity (Bakhru, Furtado, Morello, & Mathiowitz, 2013). The formulation of PNPs is a promising approach to handle these physiological obstacles and enhance the gastrointestinal absorption of drugs with limited aqueous solubility (Jung et al., 2000). The impact of PNPs' size, shape, and surface chemistry largely affects systemic drug delivery after oral administration (Malhaire, Gimel, Roger, Benoît, & Lagarce, 2016). Antiepileptic drugs can be administrated by different routes. The oral route is the classic route for chronic treatment of epilepsy, but it is challenging to be considered for the treatment of an epileptic attack. Parental, rectal, buccal, and intranasal routes represent the most common alternatives to oral administration, but each route has its advantages and limitations. Parenteral administration is the optimum solution for acute treatment due to the rapid onset of action and successful drug delivery (Musumeci, Bonaccorso, & Puglisi, 2019).

Among numerous existing polymers, poly (dl‐lactic‐co‐glycolic acid) (PLGA) has acquired the interest of several researchers due to its biodegradability, biocompatibility, sustained release profile, and maximum safety issues. PLGA is Food and Drug Administration approved for many formulations for the control of prostate and breast cancers (Crawford & Phillips, 2011; Kamaly et al., 2012). The current study is the first to adopt the formulation of PLGA NPs encapsulating ZP to investigate the bioavailability and pharmacological activity of ZP‐PLGA NPs in vivo following oral or parenteral administration.

The following study aimed to inspect the role of PNPs in enhancing the oral administration of ZP as a sedative and hypnotic drug. Besides, improving the anticonvulsant activity after parenteral administration. The effect of various formulation variables, such as the polymer amount, stabilizer concentration, and sonication time, on the characteristics of the nanoparticles, release profiles, in vivo pharmacokinetic behavior, and in vivo biological activity of encapsulated ZP were investigated in this study.

## 2 MATERIALS AND METHODS

### 2.1 Materials

PLGA (50:50) (Resomer® RG 503H, MW34 kDa), ZP, polyvinyl alcohol (PVA, 87%–89% degree of hydrolysis, molecular weight 31,000–50,000), phosphate‐buffered saline (PBS), dichloromethane (DCM), and acetonitrile were purchased from Sigma‐Aldrich Chemical Co. All other reagents were of high analytical grade.

### 2.2 Fabrication of ZP‐PLGA polymeric nanoparticles

ZP‐PLGA PNPs were formulated via a simple emulsification–solvent evaporation technique (Khan et al., 2018). To sum up, ZP and PLGA were dissolved in a common organic solvent (DCM) that can dissolve both completely. The selected organic solvent is characterized by its low boiling point, high volatility, high dissolving power, and water immiscibility. The aqueous phase compromised PVA as a stabilizer. The emulsification process was conducted via an ultrasonic homogenizer with a 3.2‐mm probe (Cole‐Parmer) to form o/w nanoemulsion. ZP‐PLGA PNPs were formed after the evaporation of DCM overnight using magnetic stirring. Finally, NPs were separated by ultra‐centrifugation at 30,000*g* for 30 min with a cooling centrifuge (Sigma Laborzentrifugen GmbH.), followed by three times of washing with ultrapure water and 2% w/v sucrose solution and lyophilized (Labconco). The final product of ZP‐PLGA NPs was kept in a desiccator at room temperature. The formulation parameters and identifier codes are listed in Table 1.

**TABLE 1:** Formulation parameters for ZP‐PLGA NPs

Formulation ID	Polymer type	Polymer concentration (% w/v)	PVA concentration (%w/v)	Sonication time (min)	Drug loaded (mg)	Organic/aqueous phase ratio
F1	PLGA	2.5	0.5	1	5	1:10
F2	PLGA	5	0.5	1	5	1:10
F3	PLGA	10	0.5	1	5	1:10
F4	PLGA	10	1	1	5	1:10
F5	PLGA	10	1.5	1	5	1:10
F6	PLGA	10	1.5	2	5	1:10
F7	PLGA	10	1.5	3	5	1:10

Abbreviations: NPs, nanoparticles; ZP‐PLGA, zaleplon‐poly (dl‐lactic‐co‐glycolic acid); PVA, polyvinyl alcohol; PLGA, poly (dl‐lactic‐co‐glycolic acid).

### 2.3 In vitro physicochemical characteristics of ZP‐PLGA NPs

#### 2.3.1 Particle size (PS) and polydispersity index (PDI)

PS and polydispersity index (PDI) were estimated with the aid of the dynamic light scattering principle (Malvern Zetasizer 5000). Briefly, 1 µl of nanosuspension was diluted at 1:10 ratio with Milli‐Q^®^ water after vortexing and sonication. Measurements were recorded as triplicates.

#### 2.3.2 Zeta potential

ZP was recorded using the same equipment of PS and PDI (Malvern Zetasizer 5000). Electrophoretic mobility was used to determine the surface charges of ZP‐PLGA NPs. Measurements were represented as triplicates.

#### 2.3.3 Surface morphology

Nanoparticles' surface morphology was observed by scanning electron microscope (SEM) on an FEI Quanta 400 FEG SEM (FEI). The samples were sited onto metal stubs and layered with a gold coat under vacuum before observation under an electron microscope.

#### 2.3.4 Entrapment efficiency

The entrapment efficiency (%E.E) of ZP was evaluated by an indirect measurement method. The supernatant containing the non‐entrapped drug was collected after centrifugation of NPs and used to quantify the free drug using the reversed‐phase high‐pressure liquid chromatography (HPLC) method (Metwally, Abdelkawy, & Abdelwahab, 2007). In detail, the HPLC system consisted of an autosampler (Waters^®^ 717), a controller (Waters^®^ 600), and a tunable absorbance UV detector (Waters^®^ 486). The mobile phase composed of acetonitrile and water (35:65%) and pumped at a rate of 1 ml/min. The amount of ZP was monitored spectrophotometrically at 232 nm. %E.E was measured by excluding the amount of free ZP from the total amount of ZP and the results were evaluated as triplicates:

(1) $$\%\;\text{Entrapment}\;\text{efficiency}\;=\;\frac{[\text{Drug}]\;\text{total}-\left[\text{Drug}\right]\text{supernatent}}{[\text{Drug}]\text{total}}\times 100$$

### 2.4 In vitro release study

The release ZP from ZP‐PLGA NPs was conducted via the dialysis process (Haggag et al., ; Haggag, Ibrahim, & Hafiz, 2020c). Four milliliters of NP suspension was put into a 5‐cm dialysis sac (spectra‐por, cut‐off 12–14 KDa). The dialysis membrane containing the NPs was immersed into 50 ml of PBS release media (pH = 7.4). The medium was stirred at 100 rpm and kept warm at 37 ± 2°C using a magnetic stirrer. After each time interval, a 1 ml withdrawn sample was collected and refilled with 1 ml of fresh PBS. The analysis of ZP concentration was performed using the previously discussed HPLC method.

### 2.5 In vivo study

#### 2.5.1 Bioavailability study

Healthy 24 male albino rabbits weighing 1.8–2 kg were used in the present study. The rabbits were held in fasting condition for 24 h previously, and the rabbits were categorized into four different groups, each containing six rabbits. Group 1 was treated orally by normal saline and served as control, whereas group 2 received a marketed ZP tablet (reference) at a dose of 1 mg·kg^−1^ (Hosny & Banjar, 2013; Noguchi, Kitazumi, Mori, & Shiba, 2004). Group 3 and group 4 were administered orally with the same dose of ZP free suspension and ZP‐PLGA NPs (F5), respectively. Two milliliters of blood samples were collected at 0, 1, 2, 3, 6, 9, 12, 15, 18, 21, and 24 h after oral intake of the drug. Samples were preserved at −20°C until analysis. Blood samples were centrifuged at 4000 rpm for 30 min with subsequent measurement of ZP concentration using the adopted HPLC method. The pharmacokinetics parameters (PK) (*C*_max_ and *T*_max_, area under curve [AUC], mean residence time (MRT), and *T*_1/2_) were measured using the plasma concentration–time curve using the WinNonlin^®^ Nonlinear Estimation Program. All experimental works were under the ethical committee of the School of Pharmacy, Tanta University, Egypt.

#### 2.5.2 Assessment of GABA level in rabbits' plasma

Along with the pharmacokinetic study, rabbits were used to measure the gamma‐aminobutyric acid (GABA) level in the plasma. After 3 h of dose administration, blood samples from all animal groups were collected and centrifuged. The plasma GABA level for each rabbit was estimated using the GABA Elisa kit (Rabbit GAMA Enzyme‐Linked Immunosorbent Assay [ELISA] Kit, Catalog No. MBS722440, MyBioSource Inc.).

#### 2.5.3 Sedative and hypnotic action

The sedative and hypnotic effect of ZP was interestingly investigated through the measurement of the effect of ZP and ZP‐PLGA NPs on sedation and hypnosis of rabbits (Bellini, Banzato, Contiero, & Zotti, 2014). Simply, the onset of sedation and hypnosis and duration of hypnosis were measured for all animal groups before starting the PK study. Regular observation for all animals was done to record the time needed for hypnosis and hypnosis duration for each animal. All experimental works were conducted in agreement with the research ethics guidelines conditioned by the Faculty of Pharmacy, Tanta University, Egypt.

#### 2.5.4 Evaluation of anticonvulsant activity

Anticonvulsant activity of free ZP and ZP‐loaded NPs was evaluated by maximal electroshock‐induced seizure (MES) model in vivo. Thirty albino rats were used for this study. Rats were classified into three groups (10 rats/group): the first group received the saline and served as the control animal group; the second group was treated with ZP suspension (20 mg/kg); and the third group received the nanosuspension of ZP‐PLGA NPs of the same dose of (20 mg/kg). Maximal electroshock (MES; 60 Hz, 0.2 s, 150 mA) was carried through auricular (ear lobe) electrodes using electroconvulsive therapy unit (Ugo Basile). The MES study was conducted 10 h following intraperitoneal injection of drug treatments. Seizure severity was scored and recorded. The evaluation was based on the extent of tonic–clonic seizure and the extent of tonic extension spread. The scoring system was reported (Wang et al., 2016). The length of tonic extension and occurrence of tonic seizures were also examined as measures of seizure severity (Wang et al., 2016).

### 2.6 Statistical analysis

PS, zeta potential, entrapment efficiency, and drug release were presented as mean ± standard deviation (SD) and treated statistically using one‐way analysis of variance (ANOVA) followed by post hoc Tukey's test. The results of in vivo studies were demonstrated as mean ± SEM and treated statistically using one‐way ANOVA followed by post hoc Tukey's test. The AUC analysis was conducted using trapezoidal rule with baseline correction. *p *< 0.05 was considered statistically significant.

## 3 RESULTS AND DISCUSSION

The current study was conducted to investigate the impact of different formulation variables on the various characteristics of the ZP nanoparticles, such as nanoparticles size, surface morphology, and entrapment efficiency. In vitro release to optimize the fabrication of ZP‐PLGA NPs having an appropriate size and high drug loading for controlled pharmacological action was investigated.

### 3.1 Influence of polymer concentration

**FIGURE 1:**
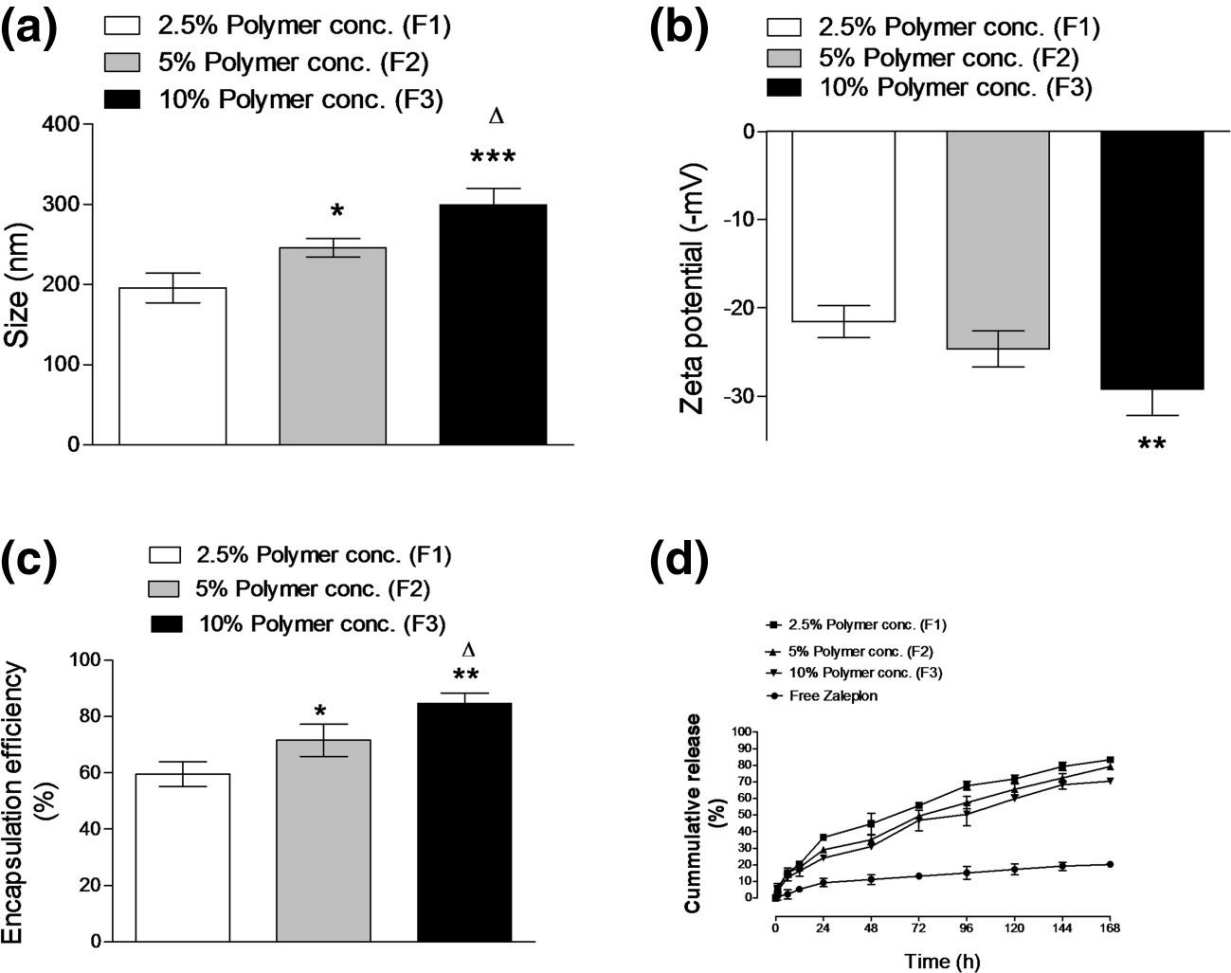
Effects of the polymer concentration on nanoparticle size (a), zeta potential (b), encapsulation efficiency (c) and zaleplon in vitro release (d). Values are mean ± standard deviation for (*n* = 3). For a–c, *p < 0.05; **p < 0.01; ***p < 0.001, compared with 2.5% w/v polymer concentration (F1). Δ*p* < 0.05 compared with 5% w/v polymer concentration (F2)

The effect of different PLGA amounts on the physicochemical characteristics of ZP‐PLGA NPs (F1, F2, and F3) was demonstrated in Figure 1. PLGA‐ZP nanoparticle sizes were significantly (*p* ˂ 0.05) increased by the increase of polymer concentration in DCM from 2.5% w/v to 5% and 10% w/v (Figure 1a). The possible reported explanation of this finding could be due to the higher viscosity of the organic phase. There is an integral correlation between PS and viscosity of both phases (aqueous and organic phases). Provided that the shear stress is a constant, an increase in the viscosity causes a considerable increase in the resistance to the exposed shear stress. Consequently, the probability of coalescence between nanoparticles during formulation is increased. The balance between agitation shear force and droplet cohesion during the processing of nanoparticles controls the size of formed emulsion droplets (Fude et al., 2005; Haggag et al., 2018a).

Concerning zeta potential, increasing the concentration of polymer to 10% w/v is followed by a significant (*p* ˂ 0.01) increase in zeta potential, which might be discussed by the intense polymer abundance on nanoparticles' surface following the increase in polymer concentration (Khan et al., 2018). The change in polymer concentration from 2.5% w/v to 5% w/v did not record a significant increase (*p* ˃ 0.05) in nanoparticles' surface charge in case of (F1, F2) (Figure 1b).

Drug encapsulation efficiency was significantly (*p* ˂ 0.05) enhanced by changing the concentration of polymer from 2.5% w/v (F1) to 5% w/v (F2) and 10% (F3) (Figure 1c). Improvement of ZP entrapment efficiency could be also attributed to the higher viscosity of the oily phase with subsequent enlargement of emulsion droplet size that created a more stable microenvironment to prevent drug escape from the organic phase to the exterior aqueous media. Another possible explanation is the rapid solidification of polymer following the increase in the polymer concentration that restricts drug diffusion from the inner oily phase (Haggag et al., 2016; Yang, Chung, Bai, & Chan, 2000).

The effect of different concentrations of PLGA on the ZP release was shown in (Figure 1d). The early burst effect was interestingly affected by changing PLGA concentrations. A significant (*p* ˂ 0.05) decline in initial burst release from 24% (F1) to 36% (F3) was detected. This may be clarified by the fast solidification process that followed due to the high polymer concentration; the polymer matrix will become condensed, resulting in smaller pores and a further tortuous assembly because of the chain entanglement, which obstructs drug transmission to release media (Yang, Chung, & Ng, 2001).

### 3.2 Influence of the PVA concentration of the aqueous phase

**FIGURE 2:**
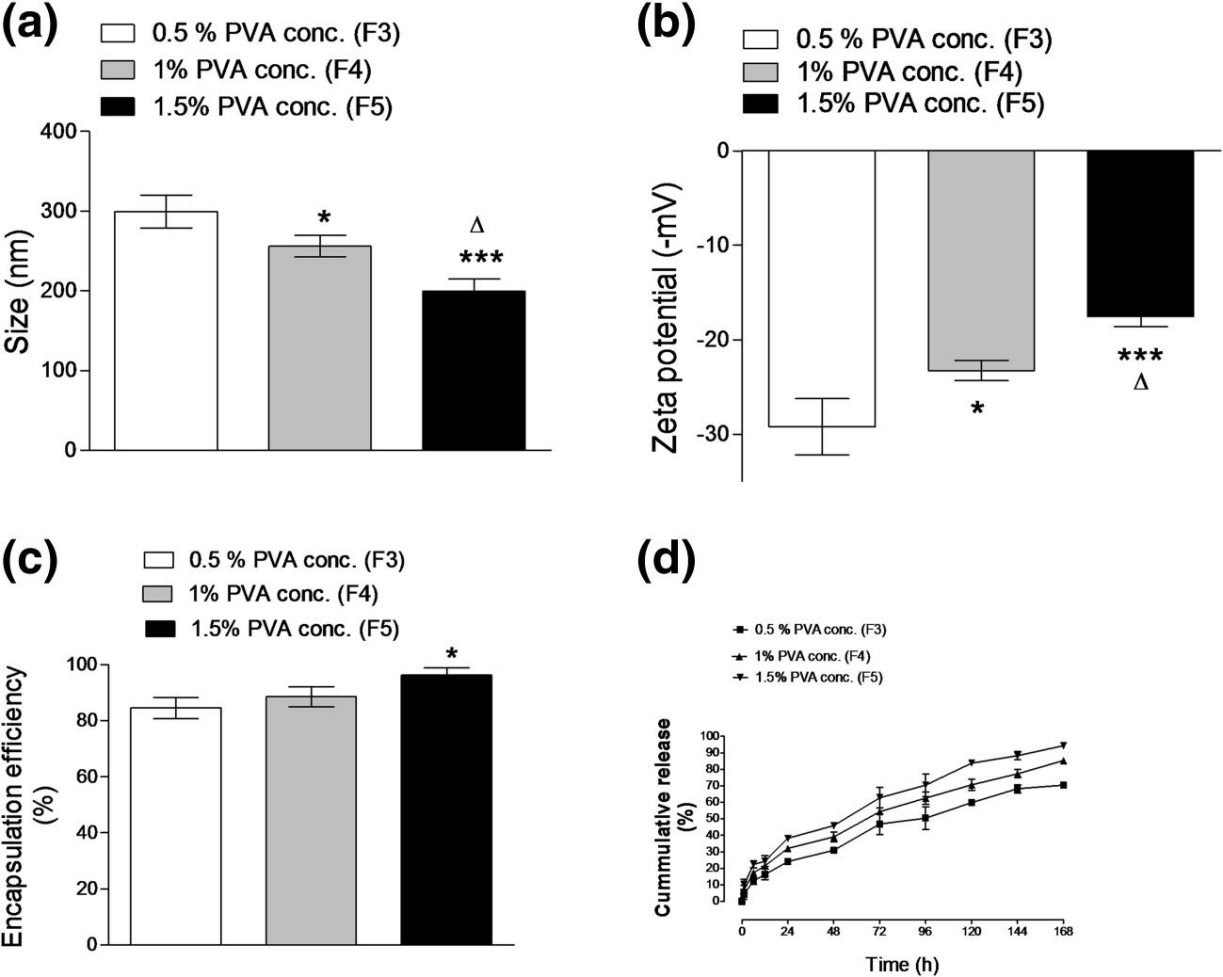
Effects of the polyvinyl alcohol (PVA) concentration on nanoparticle size (a), zeta potential (b), encapsulation efficiency (c) and zaleplon in vitro release (d). Values are mean ± standard deviation for (*n* = 3). For a–c, *p < 0.05, **p < 0.01, ***p < 0.001 compared with 0.5% w/v PVA concentration (F3). Δ*p* < 0.05 compared with 1% w/v PVA concentration (F4)

PVA is a frequently used stabilizer in the fabrication of biodegradable PNPs. PVA concentration in the aqueous phase predominately controls nanoparticles' size (Haggag et al., 2018a, 2018b; Sahoo, Panyam, Prabha, & Labhasetwar, 2002). PVA was used at three different concentrations of 0.5%, 1%, and 1.5% w/v as a stabilizer in the aqueous phase to study the effect of PVA concentration on the physicochemical characteristics of different ZP‐PLGA NPs formulations of (F3, F4, and F5) as summarized in Figure 2.

A significant (*p* ˂ 0.05) reduction in nanoparticle size of F4 and F5 was observed after altering the concentration of PVA in the external phase (Figure 2a). This was endorsed to the fact that increasing PVA concentration would raise the continuous aqueous phase viscosity and successively the stability of emulsion droplets formed during sonication. Increasing the emulsion stability in addition to the enhanced surfactant activity of PVA at higher concentrations prevented the coalescence of emulsion droplets. Therefore, smaller droplets would progressively harden to form nanospheres with a low PDI (Haggag et al., 2017; Sahoo et al., 2002).

Increasing the PVA concentration caused a significant (*p* ˂ 0.05) decrease in zeta potential. PVA concentration of 0.5% (F3) showed more intense negative zeta potential values concerning the higher PVA concentration of 1% and 1.5% w/v (F4 and F5), respectively (Figure 2b). This sharp drop in zeta potential values while increasing PVA concentrations can be explicated by the coating of nanoparticles with a residual PVA which covered the particle's charge and change the shear plane outwards from the particle surface. 0.5% PVA concentration has the least shielding effect and, consequently, more carboxyl groups are existing for ionization; therefore, the higher zeta potential was obtained (Haggag et al., 2017). Moreover, a significant (*p* ˂ 0.05) increase in ZP encapsulation and drug loading was detected after increasing the concentration of PVA in the outer water phase from 0.5% to 1.5% w/v in the case of PLGA nanoparticles (Figure 2c). This effect might be explained by two hypotheses. First, the increased viscosity of the aqueous phase minimizes the drug transport from the organic phase to the outer aqueous phase. Secondly, the higher PVA concentration resulted in a higher amount of PVA at the interface, which serves as a barrier between the organic aqueous phase that contributed to higher resistance against drug transmission out of the organic phase leading to higher drug loading (Haggad et al., 2016; Khan et al., 2017). However, increasing the concentration of PVA from 0.5% w/v to 1% w/v give rise to nonsignificant (*p* ˃ 0.05) change in encapsulation efficiency. This might be attributed to lower viscosity of 0.5% and 1% w/v PVA solutions in contrast to 1.5% PVA concentration (Sahoo et al., 2002).

Release behavior of ZP‐PLGA NPs formulated with 0.5%, 1%, and 1.5% w/v of PVA were demonstrated in (Figure 2d). The release pattern of (F3) formulated with 0.5% w/v PVA showed a significant (*p* ˂ 0.05) lower initial burst effect concerning (F4 and F5) prepared with 1% and 1.5% w/v PVA. As long as the burst release was linked to the diffusion of the surface‐attached drug, a higher PVA concentration of 1.5% resulted in smaller nanoparticles with higher surface area readily exposed to the media of release which facilitate ZP diffusion and release (Fude et al., 2005; Haggag et al., 2016).

### 3.3 Influence of sonication time

**FIGURE 3:**
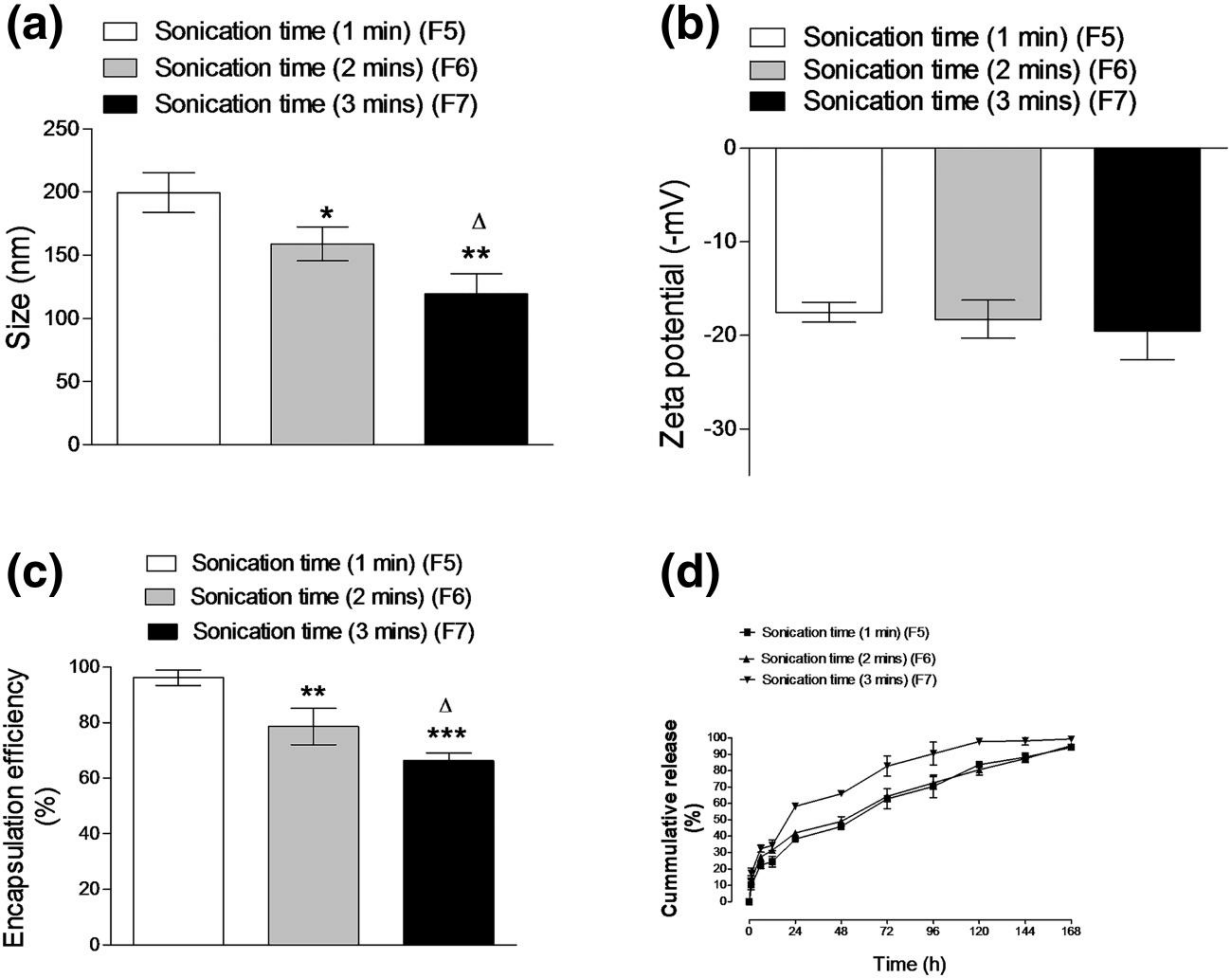
Effects of the sonication time on nanoparticle size (a), zeta potential (b), encapsulation efficiency (c) and zaleplon in vitro release (d). Values are mean ± standard deviation for (*n* = 3). For a–c, *p < 0.05, **p < 0.01, ***p < 0.001 compared with 1 min sonication time (F5). Δ*p* < 0.05 compared with 2 min sonication time (F6)

The physicochemical characteristics of three different formulations of ZP‐PLGA NPs (F5, F6, and F7) made using three different sonication times are presented in Figure 3. ZP‐loaded PLGA nanoparticles (F7) prepared by using the longest sonication time of 3 min were significantly (*p* ˂ 0.05) smaller in size than the nanoparticles (F5 and F6) prepared by using 1 and 2 min sonication time, respectively.

Increasing the sonication time produced a decrease in the PS of F7 (Figure 3a). These findings might be attributed to the higher shear stress used, which would create a suitable condition to prevent coalescence of emulsion droplets resulting in reduced emulsion droplets, which in turn led to smaller nanoparticles (Haggag et al., 2018). Increasing the sonication time from 1 min to 3 min did not count for a significant (*p* ˃ 0.05) change in nanoparticles' zeta potential (Figure 3b). However, a sharp (*p* ˂ 0.05) decrease in drug encapsulation efficiency was observed in F6 and F7 compared to F5 (Figure 3c). Increasing sonication time resulted in lower drug entrapment. This might be explained by the effect of high shear stress on polymer behavior with disruption of polymeric inner structure and PVA interfacial layer, which facilitates drug diffusion to the aqueous layer (Blum & Saltzman, 2008; Haggag et al., 2018a).

The release profile of (F5, F6, and F7) showed that the drug burst release was faster and efficiently higher from F6 and F7 which released almost 43% and 58% of ZP releases within the initial 24 h on the contrary to 38% of drug released from the F5, respectively (Figure 3d). The burst release effect is linked to the proportion of the drug that attached the nanoparticle surface (Essa, Rabanel, & Hildgen, 2010). A higher amount of drug was attached to F6 and F7 nanoparticle surfaces compared to F5 because of their smaller PS and higher surface areas. Increasing sonication time leads to the formation of a large number of pores inside the polymeric matrix and, consequently, the drug can quickly diffuse through these pores to the release medium (Bilati, Allemann, & Doelker, 2003).

Screening the previous results, we concluded that F5 showed the highest encapsulation efficiency with a small nanoparticle size of approximately 200 nm, a moderate zeta potential of −17.5 mV, the highest entrapment efficiency of approximately 96%, and a relatively low burst release of 38%. F5 was used as the optimized formula for further characterization.

### 3.4 Scanning electron microscopy

**FIGURE 4:**
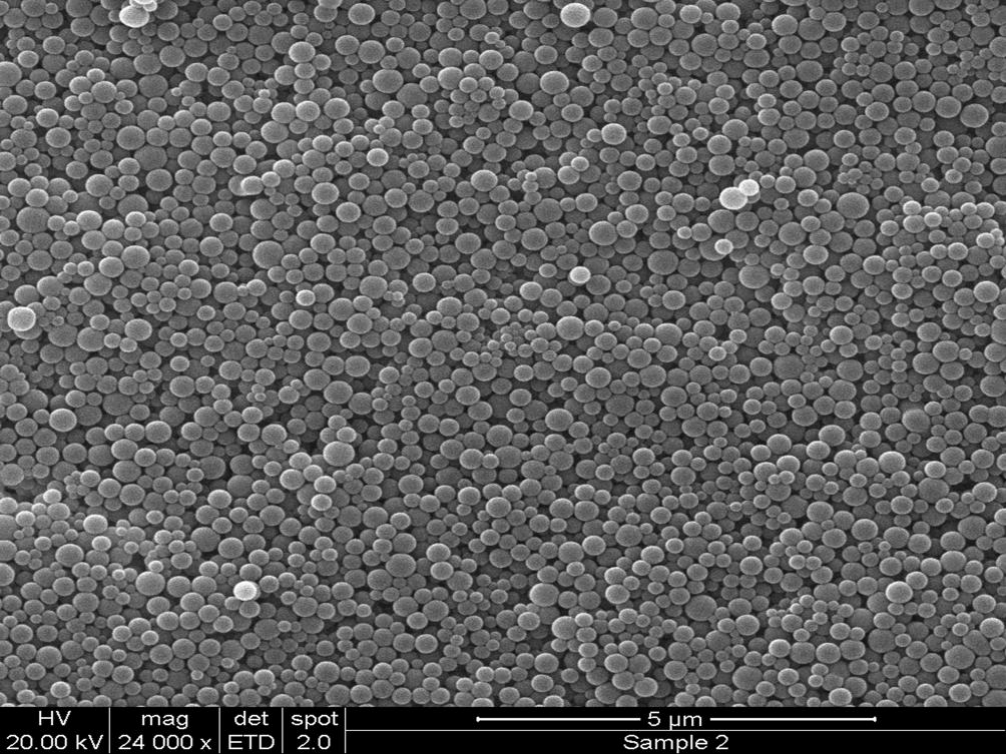
Scanning electron microscope images of zaleplon–poly (dl‐lactic‐co‐glycolic acid) nanoparticles (F5) after preparation

The SEM image of F5 was represented in Figure 4. ZP‐PLGA NPs had a smooth sphere‐shaped appearance with very low PDI. Size measurements by SEM and dynamic light scattering are highly correlated.

### 3.5 In vivo study

#### 3.5.1 Pharmacokinetic study

**FIGURE 5:**
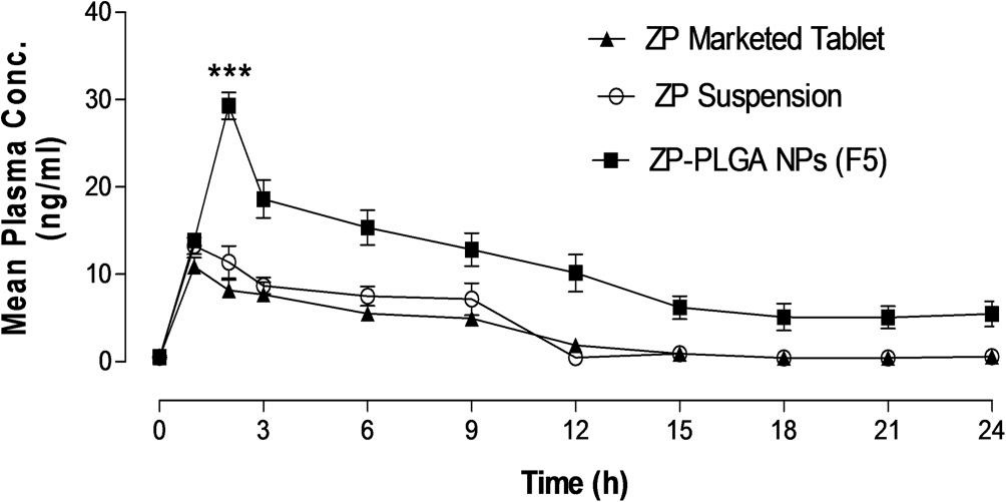
Plasma – the concentration–time curve of zaleplon (ZP) after oral administration of 1 mg/kg of different ZP formulations to rabbits. Values are mean ± SEM for (*n* = 6). ***p < 0.001 compared with ZP marketed tablet and ZP suspension. SEM, standard error mean

Zaleplon, being a class II drug, has a poor oral bioavailability as a result of limited water solubility and extensive hepatic metabolism. The mean plasma concentration versus time profiles of ZP after oral intake of ZP free suspension, ZP marketed tablet, and ZP‐PLGA NPs are demonstrated in Figure 5. The measured pharmacokinetic parameters are presented in Table 2. The pharmacokinetic study outcomes clarified that oral delivery of ZP‐PLGA NPs (F5) can efficiently alter its pharmacokinetic profile by increasing its bioavailability as compared to the marketed oral tablet and free ZP suspension.

**TABLE 2:** Pharmacokinetic parameters after oral administration of different zaleplon formulation

ZP formulation	*C*_max_ (ng/ml)	*T*_max_ (h)	AUC_tot_ (ng·h/ml)	MRT (h)	*T*_1/2_ (h^−1^)
ZP marketed tablet	10.86 ± 2.05	1.0 ± 0.0	625 ± 34.9	6.7 ± 1.38	2.14 ± 1.38
ZP suspension	13.27 ± 1.88	1.0 ± 0.0	774 ± 65.6	7.9 ± 2.34	2.79 ± 1.89
ZP‐PLGA NPs (F5)	29.31 ± 3.08^***^	2.0 ± 0.0	2135 ± 345^***^	13.75 ± 3.48^***^	8.25 ± 3.19^***^

*Note*: Values are mean ± SEM for (*n* = 6).

Abbreviations: AUC, area under curve; MRT, mean residence time; NP, nanoparticle; ZP‐PLGA, zaleplon–poly (dl‐lactic‐co‐glycolic acid).

^***^ *p* < 0.001 compared with ZP marketed tablet and ZP suspension.

Following oral administration, plasma concentrations of ZP‐PLGA NPs were all significantly (*p* ˂ 0.05) advanced than that of the free drug and marketed tablet at every time point. Plasma concentration–time curve showed the *C*_max_ for ZP‐PLGA NPs (29.31 ± 3.08 ng/ml) was significantly (*p* ˂ 0.001) higher concerning marketed ZP tablet (10.86 ± 2.05 ng/ml) and free drug suspension (13.27 ± 1.88 ng/ml). However, the time to achieve the maximum plasma concentration (*C*_max_) was shorter for marketed ZP tablet and ZP suspension compared to that of ZP‐PLGA NPs. The total AUC, which represent the extent of ZP absorption, was additionally significantly (*p* < 0.001) higher for ZP‐PLGA NPs (F5) (2135 ± 345 ng·h/ml) compared to marketed ZP tablet (625 ± 34.9 ng·h/ml) and ZP suspension (802 ± 65.6 ng·h/ml). The MRT and *T*_1/2_ were more pronounced for F5 due to the slower elimination of ZP from drug‐loaded PLGA NP formulation.

ZP‐PLGA NPs formulation exhibited a 3.42‐fold enhancement in the oral bioavailability as compared to marketed ZP tablet and a 2.75‐fold increase over ZP suspension. The PK result illustrated that Z‐PLGA NPs showed higher plasma concentration, lower clearance, and a longer half‐life in comparison to ZP marketed tablet and ZP suspension in rabbits. The smaller nanoparticle size of ZP‐PLGA NPs with subsequent higher effective surface area, which further leads to increased extent and duration of contact with gastro‐intestinal mucosa with subsequent improvement of rate and extent of drug absorption (Du et al., 2018). The formulation of this drug as a nanosystem augmented its solubility and permeability (Xie et al., 2011). In addition, PLGA NPs can reach systemic circulation through gut‐associated lymphatic transport, and thus minimizes the hepatic metabolism of the drug (Ahmad et al., 2015). The greater the lipophilicity of nanosystems, the higher the extent of lymphatic transport. These hypotheses, individual and/or in combination, could have donated to the optimization of the bioavailability of ZP from PLGA NPs (Dahan & Hoffman, 2008).

#### 3.5.2 Assessment of plasma GABA level

**FIGURE 6:**
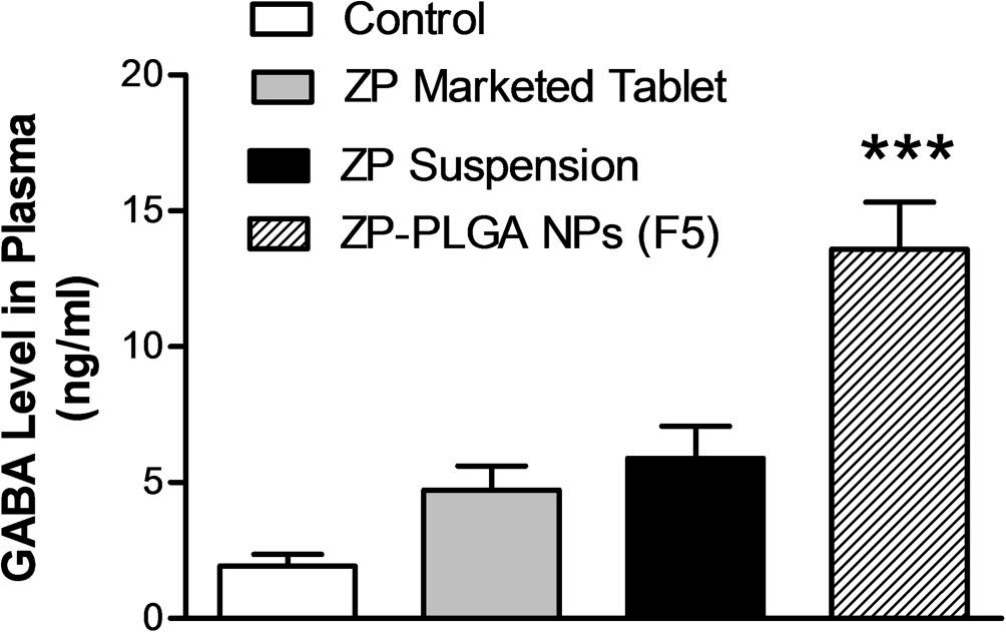
Plasma level of gamma‐aminobutyric acid after oral administration of 1 mg/kg of different Zaleplon (ZP) formulations to rabbits. Values are mean ± SEM for (*n* = 6). ***p < 0.001 compared with ZP marketed tablet and ZP suspension

The pharmacological activity of ZP depends upon its mechanism of action on (GABA‐A) receptors in the brain which increased the GABA concentration (Sanger, 2004). Herein, the plasma GABA level of different animal groups receiving different ZP formulations was measured using a specific rabbit ELISA kit, and the results were represented in Figure 6. The average plasma GABA level of animals treated with ZP‐PLGA NPs (F5) was significantly (*p* ˂ 0.001) higher concerning the GABA level of animals treated with either ZP suspension or ZP marketed tablet. The results confirmed the superior biological activity of ZP‐PLGA NPs compared to ZP suspension and ZP marketed tablet. This might be attributed to better systemic absorption and higher oral bioavailability of drug‐loaded NPs compared to other ZP formulations.

#### 3.5.3 Assessment of sedative and hypnotic effect

**FIGURE 7:**
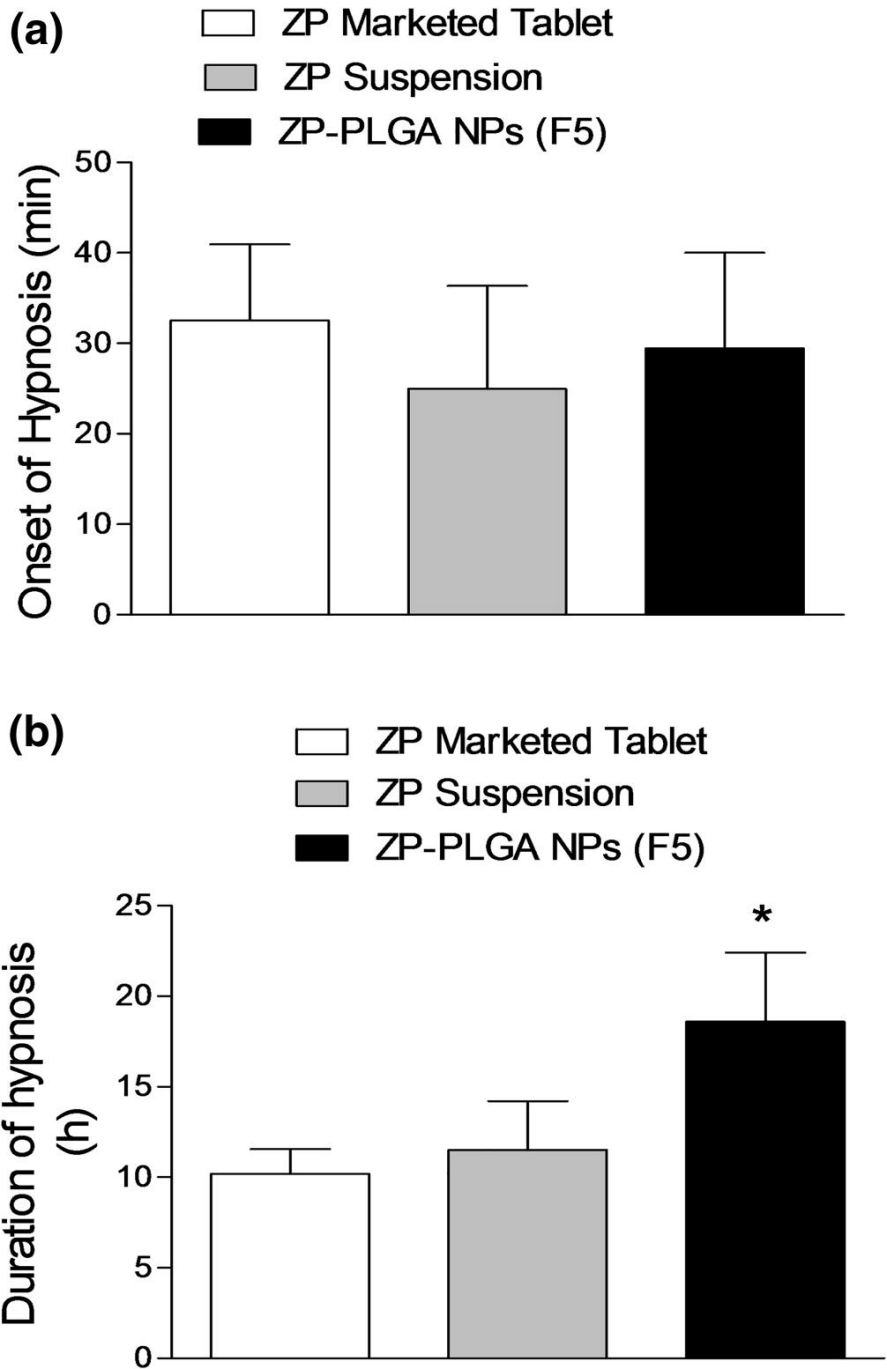
Evaluation of sedative and hypnotic effect oral administration of 1 mg/kg of different Zaleplon (ZP) formulations to rabbits; (a) onset of hypnosis; (b) duration of hypnosis. Values are mean ± SEM for (*n* = 6). *p < 0.05 compared with ZP marketed tablet and ZP suspension

ZP is used for the treatment of insomnia due to its agonist effect on the GABA receptor as it indorses sleeping by enhancing the effect of GABA as an inhibitory neurotransmitter (Sanger, 2004). The average onset and duration of hypnosis were measured for animal groups treated with different ZP formulations and the results were demonstrated in Figure 7. Animals treated with different ZP formulations showed comparable onset of hypnosis (*p* ˃ 0.05) (Figure 7a). However, animals treated with ZP‐PLGA NPs exhibited significantly (*p* ˂ 0.05) prolonged duration of hypnosis compared to other ZP formulations (Figure 7b). The hypnotic action of ZP lasted for longer duration in the rabbits treated with NPs formulations due to sustained release of ZP from PLGA NPs, which resulted in longer drug residences in the systemic circulation (Dudhipala & Janga, 2017). The sedative and hypnotic effects of different ZP treatments were highly agreed with our previous results of plasma GABA levels. Animals treated with ZP‐loaded NP showed a prolonged sleep duration due to high plasma GABA level compared to animals treated with ZP suspension or ZP‐marketed tablet which showed significantly lower GABA level and thereby shorter sleep duration.

#### 3.5.4 Evaluation of anticonvulsant activity

**FIGURE 8:**
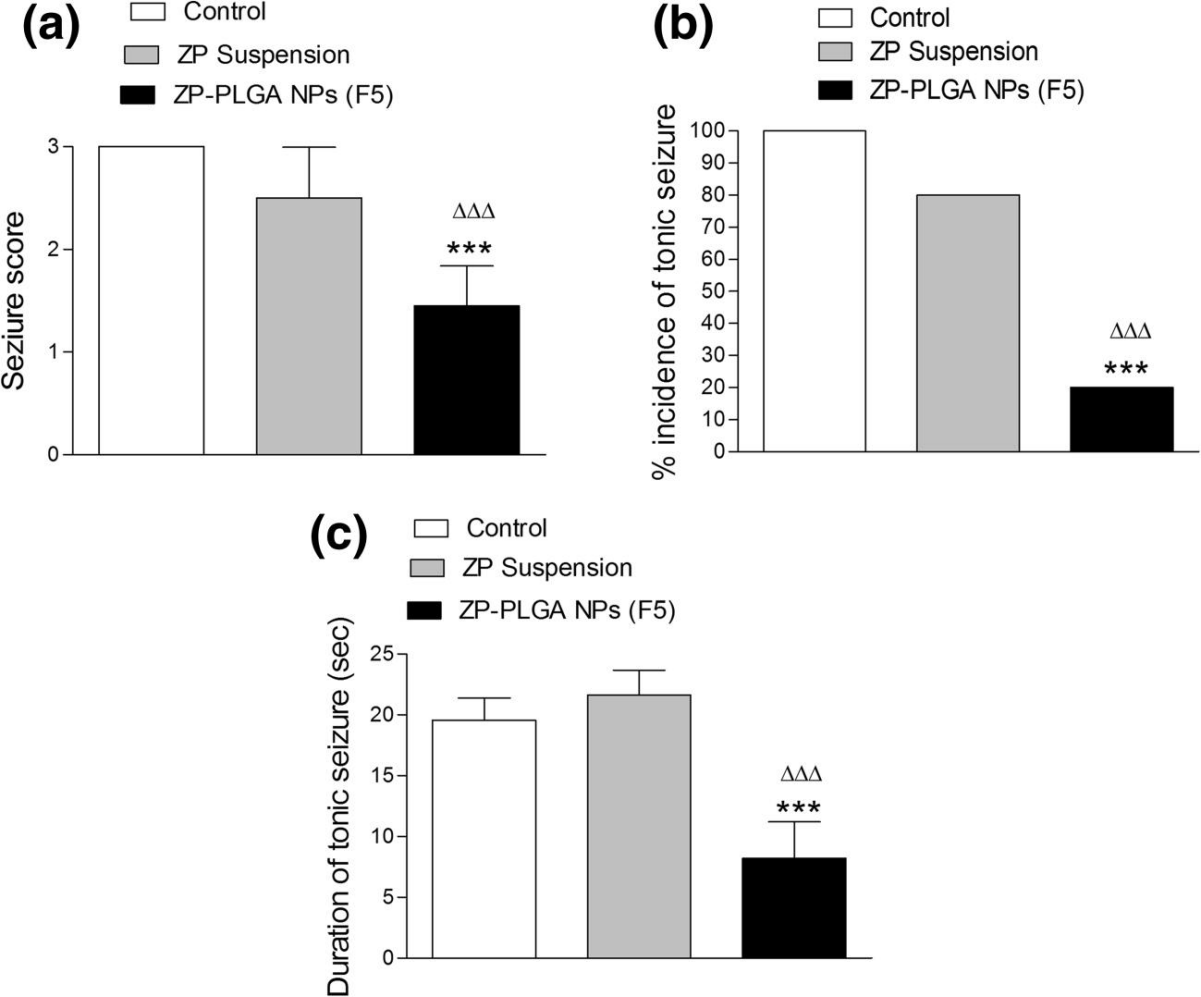
Evaluation of anticonvulsant activity after intraperitoneal administration of 20 mg/kg of different zaleplon (ZP) formulations to rats: (a) seizure score; (b) % incidence of tonic seizure; and (c) duration of tonic seizure. Values are mean ± SEM for (*n* = 10). ***p < 0.001 compared with control and ΔΔΔ *p* < 0.001 compared with ZP suspension

The MES model is used for generalized tonic–clonic seizures. This epilepsy model was used to show the efficacy of antiepileptic agents against partial and generalized seizure types. The MES screening tool is useful because it can provide a quick prediction of the anticonvulsant activity of tested drugs with minimal investment and experience (MareŠ & KubovÁ, 2006). In vivo results were represented in Figure 8. A high seizure score was observed for animals treated with free ZP, which was nonsignificant from the control group (*p* ˃ 0.05) (Figure 8a). A significant decrease in the stage of seizure was observed in the case of animals that received ZP‐PLGA NPs (*p* ˂ 0.001). The incidence of tonic convulsion was represented for each group (Figure 8b). The number of rats showed tonic seizures/total rats used and % incidence was calculated for each group of rats. It is clear that ZP‐PLGA NPs exhibit a significant decline (*p* ˂ 0.001) in the number of convulsed rats with subsequent improvement in % incidence compared to ZP free suspension. Moreover, a marked decrease in the duration of tonic seizures (*p* ˂ 0.001) was observed for animals treated with drug‐loaded NPs compared to control and animal treated with free ZP (Figure 8c). This augmented antiepileptic effect of ZP‐PLGA NPs may contribute to the prolonged systemic circulation and sustained drug release from ZP‐PLGA NPs, which sustains its pharmacological effect. On the contrary, ZP free suspension is rapidly eliminated from the blood due to its very short elimination half‐life.

## 4 CONCLUSIONS

The novelty of this study is the usage of biocompatible PLGA PNPs for oral and parenteral administration of ZP, and in vivo evaluation of its pharmacokinetic behavior and pharmacological activity which is being documented for the first time. Optimizing the physicochemical properties of ZP‐PLGA NPs can be achieved through adjusting different process variables, especially the polymer concentration, stabilizer concentration, and time of sonication. Optimum ZP‐PLGA NPs showed a small size of 200 nm and a high entrapment efficiency of 96%. ZP‐PLGA NPs exhibited a significant increase in oral bioavailability represented by higher *C*_max_ and AUC values compared to ZP suspension and ZP marketed tablet. Optimum ZP‐PLGA NPs showed significantly longer MRT and *T*_1/2_ compared to other ZP formulations. The bioavailability of ZP was increased by more than threefold compared to ZP marketed tablet. Furthermore, in vivo results reinforced the enhanced biological activity of ZP‐PLGA NPs represented by its superior sedative and hypnotic effect compared to the conventional dosage form. The parenteral administration of ZP‐loaded NPs exhibited a potent anticonvulsant activity concerning the free drug. In conclusion, our results indicate that the ZP‐PLGA nanoparticulate system is a hopeful formulation strategy for the improvement of ZP oral and parenteral administration.

## CONFLICT OF INTEREST

The authors declare no financial or personal relationships with other people or organizations that could inappropriately affect this study. There are no competing interests.
